# OptoDyCE as an automated system for high-throughput all-optical dynamic cardiac electrophysiology

**DOI:** 10.1038/ncomms11542

**Published:** 2016-05-10

**Authors:** Aleksandra Klimas, Christina M. Ambrosi, Jinzhu Yu, John C. Williams, Harold Bien, Emilia Entcheva

**Affiliations:** 1 Department of Biomedical Engineering, Stony Brook University, Stony Brook, New York, USA; 2 Present address: Department of Biomedical Engineering, George Washington University, Washington, DC, USA

## Abstract

The improvement of preclinical cardiotoxicity testing, discovery of new ion-channel-targeted drugs, and phenotyping and use of stem cell-derived cardiomyocytes and other biologics all necessitate high-throughput (HT), cellular-level electrophysiological interrogation tools. Optical techniques for actuation and sensing provide instant parallelism, enabling contactless dynamic HT testing of cells and small-tissue constructs, not affordable by other means. Here we show, computationally and experimentally, the limits of all-optical electrophysiology when applied to drug testing, then implement and validate OptoDyCE, a fully automated system for all-optical cardiac electrophysiology. We validate optical actuation by virally introducing optogenetic drivers in rat and human cardiomyocytes or through the modular use of dedicated light-sensitive somatic ‘spark' cells. We show that this automated all-optical approach provides HT means of cellular interrogation, that is, allows for dynamic testing of >600 multicellular samples or compounds per hour, and yields high-content information about the action of a drug over time, space and doses.

## Abstract (web summary)

The efficiency of preclinical drug testing and characterization of cellular function can be improved through the use of optogenetic tools. Here Klimas *et al*. present and validate OptoDyCE, a fully automated system for all-optical high-throughput cardiac electrophysiology.

## Graphical abstract

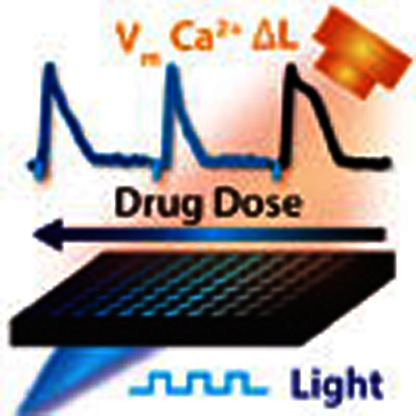

The development of new drugs is lengthy and inefficient, where the approval process alone takes on average 7–10 years (ref. 1). In the United States, <0.05% of all compounds undergoing preclinical tests become marketed drugs, and <30% of compounds evaluated in clinical trials make it to market^2^. Perhaps most costly, and with greatest negative societal and ethical impact, is the withdrawal of drugs from the market after they have been approved. Insufficient or inadequate tools for predicting failure before more expensive phases of testing both in animal and human, drive up the drug costs and decrease the desire for pharmaceutical companies to pursue more ‘high-risk' drugs that would result in little payout. In 2004, it was estimated that a 10% improvement in failure prediction before clinical trials could save $100 million in development costs per drug^3^. Ultimately, developing tools for improved failure prediction of a drug in earlier stages of the development process is necessary to reverse current trends. In the last 40 years, over 20% of drugs discontinued at all phases of development, including discovery, preclinical and clinical evaluation, and post-market surveillance has been due to cardiac toxicity, where unintended interactions with cardiac ion channels result in pro-arrhythmic effects^4^. In response, international regulatory agreements were developed that mandate testing of all new drugs, both cardiac and non-cardiac, for cardiac liability, including drug-induced long QT interval (LQT) and risk for development of life-threatening arrhythmias, such as Torsade de Pointes (TdP)^5^.

Currently required preclinical cardiotoxicity testing (part of the drug development process, Fig. 1) specifically focuses on a drug's blocking action on the hERG K^+^ channel, which provides one of the main repolarizing currents in cardiomyocytes. The blocking of this channel impacts repolarization and is often associated with LQT and thus, with increased risk of TdP. However, it has recently been recognized that a drug's pro-arrhythmic effect, or ‘torsadogenicity', is often shaped by its action on multiple ion channels, whereas the net effect may be different than the outcome of a simple HERG K^+^ channel block^5^,^6^,^7^,^8^,^9^. Indeed, there are hERG K^+^ blockers that are known to not cause TdP (for example, ranolazine or verapamil), resulting in false positives by the current testing methodology. Similarly, drugs with minor effect on the hERG K^+^ channel but causing TdP (for example, tedisamil), produce false negatives^7^. As a result, an integrative (both cell-level and multicellular) view is essential, and current regulations need to be revisited (see Supplementary Fig. 1 overviewing the Comprehensive *in Vitro* Pro-arrhythmia Assay^5^ Concept). Computational efforts are underway^7^,^9^ to integrate multichannel data obtained in recombinant expression systems (non-myocytes) to predict the action of a drug on the human cardiac action potential (AP; see Supplementary Fig. 1). While computational models are powerful in simulating a wide range of conditions, they still require validation, and heavily rely on the extensive experimental data for individual ion channels. In addition, these data have their limitations due to being obtained in non-myocytes and by non-high-throughput (HT) technology. Importantly, this type of experimental data (patch clamp data on select ion channels, Supplementary Fig. 1) still leave the computational models underconstrained. This high level of uncertainty results from the missing detailed information on calcium and contractility handling, as well as important intracellular-signalling aspects. For example, models incorporating even an extensive ion channel data set, obtained using patch clamp in non-myocytes, may not be able to predict the pro-arrhythmic effects of a leukaemia drug such as ponatinib, a tyrosine kinase inhibitor, or other non-classic multi-target regulators of electrophysiology. An alternative, more direct and relevant experimental testbed for drug/cardiotoxicity screening may be provided by direct cell-level measurements in cardiomyocytes. In particular, human patient-derived cardiomyocytes (induced pluripotent stem cell-derived (iPSC-CMs)) show great potential, considering recent great strides in their optimization and production-scaling^10^,^11^. The use of iPSC-CMs provides an exciting venue for patient-specific drug testing, as acquiring native human heart tissue from the patient is undesirable and not scalable for use in HT technology (Fig. 1). The functional data obtained in myocytes by HT technology will not only yield an independent risk assessment of a drug on human cardiac electrophysiology, but will also help improve and constrain computational models developed in this area. However, there are currently no HT solutions (ability to screen >10,000 compounds a day) for performing robust cardiomyocyte electrophysiological testing.

Classic electrophysiology involves physical contact and therefore is inherently very low throughput (manual). New technical developments towards increased throughput^12^,^13^ include the automated planar patch, IonWorks by Molecular Devices, at the single-channel level; the Fluorometric Imaging Plate Reader (FLIPR) by Molecular Devices; Multichannel Electrode Arrays recording local field potentials by Axion Biosystems; impedance-based assays with xCELLigence by Acea Biosciences; and the kinetic plate reader FDSS/μCell by Hamamatsu for cellular measurements (see Supplementary Table 1 for a detailed comparison). The following limitations of these systems motivate the need for further developments towards HT cell-level electrophysiology: (1) Requirements for contact prevent scaling to the HT-level—a non-contact modality is a must. Examples of contact-requiring systems include IonWorks, Multichannel Electrode Arrays and xCELLigence. (2) Lack of electrophysiologically relevant fast readout prevents tracking of fast APs. FLIPR and the FDSS/μCell system, using optical sensing for cellular measurements, are highly parallel, but are unable to capture AP morphology with high temporal resolution. (3) Inability for dynamic actuation (pacing with adaptable protocols) and frequency-response testing (for example, FLIPR), which is quite relevant in drug-induced cardiotoxicity^14^. (4) Cell-type restrictions: more phenotypic outputs, such as iPSC-CMs or primary CMs as testbeds, are desirable rather than the currently used recombinant expression systems. However, handling limitations present challenges (for example, in IonWorks, a proper seal can be reliably formed only with ‘well-behaved' cell lines^12^). (5) None of the current automated systems can characterize tissue-level/multicellular effects, in two- or three-dimensional (2D or 3D, respectively), even though arrhythmias are inherently spatio-temporal phenomena. An all-optical electrophysiology approach^15^,^16^,^17^ can overcome these limitations and facilitate HT-level cellular testing through built-in parallelism. The contactless optical stimulation and readout can be used over millions of locations simultaneously, including 3D settings. Such a system has not been realized to date.

Here, we present OptoDyCE, the first fully automated platform for all-optical dynamic interrogation of cardiomyocyte electrophysiology. By using optogenetic tools, we are able to perform dynamic interrogation of multiple cell types, including human iPSC-CMs in monolayers or in small 3D cell constructs, with applicability to drug testing. While OptoDyCE can work with various experimental systems, its combination with scalable (HT-compatible) models, like stem cell-derived cardiomyocytes and small engineered cardiac syncytia, is particularly relevant to the drug screening and testing process (Fig. 1). We demonstrate the HT capabilities of OptoDyCE using multicellular samples in 96-well format by combining optogenetic actuation with simultaneous optical sensing of voltage, intracellular calcium or contractility by synthetic red-shifted dyes or dye-free videotracking. OptoDyCE permits the fast quantification of cardiomyocytes' electrophysiological and electromechanical response to a drug over time and space at both the cellular and global scale, to an extent that has not been achieved by any other system to date.

## Results

### Considerations for applying optogenetics to drug testing

New fast optogenetic tools for optical stimulation (actuation)^18^,^19^,^20^ and recording (sensing)^17^,^21^,^22^ offer attractive solutions for the observation and fine control of multiple cells simultaneously, but their limitations must be carefully considered in the context of drug screening (Fig. 2). Both optogenetic actuators and sensors are biological entities that contain essential elements of ion-channel proteins, making them potentially susceptible to the drugs being tested. We consider the extent of such undesirable effects on the electrophysiological response of the cells of interest. The action of a fast optogenetic actuator, for example, Channelrhodopsin-2 (ChR2), which provides only a short stimulation impulse in cardiomyocytes can be viewed as time-detached from the electrophysiological response (Fig. 2a), hence mostly benign. Indeed, we show computationally that for brief light pulses, even marked hypothetical drug effects on the ChR2 current amplitude and/or kinetics are practically inconsequential for the optically triggered APs and calcium transients (CTs), as long as light irradiances are maintained at supra-threshold levels relative to what is required to activate ChR2 (Fig. 2b–f, for details see the subsection ‘Computational Analysis of Optogenetics and Cell Coupling' in the ‘Methods' section). In contrast, an optogenetic sensor, for example, the voltage sensor VSFP2.3 ref. 23, is continuously engaged and thus fully temporally convolved with the electrophysiological response (Fig. 2a). Even a mild drug action on the sensor can profoundly alter the electrophysiological readout (see computational predictions, Fig. 2g). The same applies to other voltage^17^,^22^ or calcium (GCaMP) optogenetic sensors, even if they exhibit superior kinetics compared with VSFP2.3. While great for long-term monitoring (order of days-to-months)^22^, channel-based optogenetic sensors may not be ideal for acute (order of minutes-to-hours) drug-testing applications due to such potential direct interference; instead, classic synthetic optical dyes for voltage and calcium or dye-free imaging may be more suitable, as they are already used in industrial applications.

### The OptoDyCE system for cardiac electrophysiology

We describe here OptoDyCE, an automated system for all-optical dynamic cardiac electrophysiology testing at the cellular/multicellular level, which combines optogenetic actuation via ChR2 with simultaneous optical sensing of voltage or intracellular calcium by synthetic red-shifted dyes (di-4-ANBDQBS and Rhod-4AM, respectively) spectrally compatible with ChR2, or dye-free video-tracking of contraction. The HT capabilities of OptoDyCE are illustrated with multicellular samples in 96-well format (Fig. 3). For several decades, optical techniques, including video recording of mechanical contractions and optical imaging of APs and CTs, obtained by using synthetic dyes and more recently, by optogenetic probes, have provided vital understanding of cardiac electrophysiology. However, their integration with optical pacing (for all-optical electrophysiology^17^,^24^) is a key development for cardiotoxicity testing because of the frequency-dependent aspect of drug actions and arrhythmia predictions^14^. Therefore, parallelism/scalability in both stimulation and recording are crucial to increasing throughput when testing a dynamic system like cardiac tissue for instabilities (arrhythmias).

In OptoDyCE, contactless optical pacing reliably triggers voltage (*V*_m_) and calcium ([Ca^2+^]_i_) signals, as well as quantifiable mechanical contractions, in either neonatal rat ventricular myocytes or iPSC-CMs in both cell monolayers and 3D structures (experimental data in Fig. 3a,b,e; Supplementary Figs 2–3). The ability for optical pacing is imparted via one of two quick and efficient transduction methods applied within 24–48 h before experimentation to yield: (1) OptoHTS: using direct adenoviral gene delivery in human (ChR2-hiPSC-CM) or neonatal rat ventricular cardiomyocytes (ChR2-CM)^15^,^25^,^26^; or (2) sOptoHTS: ‘sprinkling' of dedicated light-sensitive ‘spark' cells on top of samples of non-transduced cardiomyocytes, a version of our ‘tandem-cell-unit' concept^27^ (Fig. 3c–e; for details see the subsection ‘Gene and Cell Delivery of Optogenetic Actuation' in the ‘Methods' section).

We validate OptoHTS by comparing AP and CT morphology of optically stimulated ChR2-CM samples and electrically paced non-transduced CM samples, confirming that optogenetic pacing is a suitable alternative to electrical stimulation for drug testing purposes (Fig. 3f,g), as predicted computationally^25^,^28^. sOptoHTS provides a more attractive, modular method of light sensitization: a bank of generic ‘spark' cells (light-sensitized somatic cells, including immortal cell-lines) can be used in conjunction with a variety of non-modified experimental cardiac systems. Eliminating the need for genetic transformation of the target cells, and the associated efforts for optimization of gene delivery in each studied cell type, constitute significant advantages of sOptoHTS. However, caution should be applied regarding the geometry of the ‘spark' cell distribution, since loading effects of higher ‘spark'-cell concentrations can locally shorten the AP (Fig. 3h, Supplementary Fig. 4), while still having minimal effects on CT morphology. Proper ‘spark'-cell delivery, such as a localized/patterned pacing site^29^, can easily address the issue.

A fully automated HT version of OptoDyCE in 96-well format is demonstrated here using an optical setup, custom-built around an inverted microscope using a high-speed camera, an automation protocol and a custom-developed software for semi-automated analysis (Fig. 3i,j, Supplementary Fig. 8). In the current proof-of-concept implementation of OptoDyCE, dynamic drug-dose testing using a multi-beat pacing protocol can be performed on a 96-well platform in <10 min (Fig. 4a, Supplementary Fig. 5). High spatio-temporal resolution video recordings obtained by the system in these 10 min provide over 30,000 single-cell readouts per 96-well plate, probed by multiple pacing stimuli. These records can be investigated at both the global and cellular scale to assess pro-arrhythmic risk by quantifying shape and duration parameters of the voltage, calcium or contraction responses, and also sub-cellular spontaneous Ca^2+^ release (SCR) events, instabilities in intracellular calcium (Supplementary Fig. 6), abnormalities in the AP morphology, for example, early afterdepolarizations (EADs; Supplementary Fig. 7), as well as abnormal mechanical activity (aftercontractions; Supplementary Fig. 3). The system has been designed to be easily and economically adopted; compared with a prior report on all-optical electrophysiology in neurons^17^, we use low-power LED light sources and portable, modular components allowing straightforward customization.

### Drug-dose–response testing using OptoHTS

To validate the OptoDyCE system as well as illustrate the range of dynamic information that can be obtained, we chose the well-understood class-IV antiarrhythmic agent nifedipine to perform drug-dose–response testing. The drug was applied in 12 doses (0–50 μM) to 96 ChR2-CM samples (Fig. 4a). Using optical pacing at 1 Hz, we quantified the dose–response to nifedipine in terms of AP duration (APD) and CT duration (CTD; Fig. 4b–g). Expected APD shortening (Fig. 4b–d) and CTD shortening (Fig. 4e–g) were observed, especially at the plateau phase (APD25/CTD25 and APD50/CTD50), due to nifedipine blocking the inward L-type calcium current, *I*_CaL_. Nifedipine caused CTD to monotonically decrease up to 10 μM (Fig. 4f,g). In contrast, after maximum APD shortening at around 1 μM, corresponding to maximum block of *I*_CaL_ reached at that concentration (Fig. 4d, inset), the APD response to nifedipine reversed its direction, as seen clinically^8^. This is likely due to indirect (voltage-mediated) or non-specific action on other ion channels, partially countering the *I*_CaL_ block (Fig. 4d). Note that the benefits of our *in vitro* HT platform are in the ability to quickly and finely probe many concentrations, and to help determine the ‘therapeutic window', that is, the window for which a drug is both effective (has the desired action) and safe. Clinically, the drug-metabolizing action of the cytochrome P450 enzymes present in cells can amplify or suppress the effect of a drug resulting in a lower or higher apparent drug dose (as in some failed drugs^2^); our data can be used to judge the ‘room for error' in the therapeutic window for a drug.

### Validation of functional drug testing using sOptoHTS

The development of sOptoHTS using dedicated ‘spark' cells was motivated by the search for a very simple and quick solution for optical actuation without genetically modifying the target cells (cardiomyocytes) under investigation. The genetic modification of primary cardiomyocytes or iPSC-CMs to make them light-sensitive in OptoHTS requires optimization that may be cell type or clone specific. In contrast, a stable ‘spark' cell line is an attractive solution for industrial applications as it does not require any development on the user end; it can be provided as a simple reagent to be added (‘sprinkled') shortly before experimentation (24 h); and a variety of somatic (non-excitable) cells can serve as donor ‘spark' cells for optical actuation, including cardiac fibroblasts.

Seeking validation for sOptoHTS, we further compared the dose-dependent effects of nifedipine and of dofetilide using the two methods, OptoHTS versus sOptoHTS (Fig. 5). Dofetilide, a class-III anti-arrhythmic agent and intended hERG channel blocker, has a known risk for drug-induced LQT and TdP due to its APD-prolonging action^8^, making it a suitable choice for validating the system's ability to discern both APD shortening (nifedipine) and APD prolongation (dofetilide) for use as a drug-testing platform. sOptoHTS was able to successfully track the drug-dose-dependent effects on APD and CTD, similar to OptoHTS (Fig. 5a–d) and using standard tandem-cell-unit co-cultures (Supplementary Fig. 9). With proper tuning of the ‘spark' cell distribution, this simple and truly modular approach provided by sOptoHTS can be reliably applied to HT electrophysiological drug testing.

### Dynamic functional probing over time and space by OptoDyCE

Electrophysiological responses are frequency dependent, therefore passive observation of spontaneous activity^22^ is generally insufficient in drug testing and for arrhythmia assessment. Unlike most currently used systems (Supplementary Table 1), our platform allows for active dynamic interrogation, such as robust pacing protocols that can reveal *V*_m_, [Ca^2+^]_i_ or contraction's frequency response (restitution) and temporal instabilities (Fig. 6a, Supplementary Figs 3,6 and 7). For example, a consistent generation of voltage instabilities known as alternans can be captured at 2 Hz optical pacing in the presence of 2 μM dofetilide due to drug-induced APD prolongation (Fig. 6b).

Restitution and temporal or spatial variability assessed by median absolute deviation (MAD; see for details see the subsection ‘Data Processing and Analysis' in the ‘Methods' section) can be quantified as function of drug dose (Fig. 6c–h). These are directly relevant to the ‘torsadogenecity' of a drug, providing a much more complete assessment than traditional (single-channel block) testing or current state-of-the-art assays (Supplementary Table 1). Our dynamic testing data reveal that nifedipine action on peak calcium (per cent change) is dose-dependent (*P*<0.05 obtained using an analysis of variance (ANOVA) test followed by a Tukey–Kramer *post hoc* correction for multiple comparisons) but frequency-independent (Fig. 6c,d). Furthermore, nifedipine reduces temporal variability of peak calcium (assessed by MAD), and this reduction is augmented by higher frequency pacing (Fig. 6e). For dofetilide, we found enhanced relative APD50 prolongation at higher frequency, which is opposite to purported reverse-use dependence (Fig. 6f,g). Triggered pro-arrhythmic events resulting in drug-dose-related temporal variations in AP morphology, including dose-dependent increase in EADs, can be revealed in optically paced samples treated with dofetilide (Supplementary Fig. 7). Furthermore, because of the ability to study multicellular samples, spatial variability can be quantified as a function of drug dose by analysing individual cells or regions of interest within the same sample per well (Fig. 6h, see also Supplementary Figs 5 and 6). For example, we found that dofetilide at 2 μM increases spatial variability in APD (that is, increases dispersion of repolarization—a known pro-arrhythmic factor), compared with control during 1-Hz pacing (*P*<0.05 for APD50 obtained using ANOVA test followed by a Tukey–Kramer *post hoc* correction for multiple comparisons). Dispersion of repolarization and abnormal AP events (for example, EADs), in addition to being caused by heterogeneous block of the delayed-rectifier K^+^ channel (on application of dofetilide), can also be linked to localized SCR—a recognized factor in the development of life-threatening arrhythmias, including drug-triggered events and a hallmark of heart failure^30^. By recording multicellular samples with cellular-level resolution, we are able to identify SCR events, triggered by a drug, which may not be observed in the global traces (Supplementary Fig. 6). Most of the electrophysiological results presented here can be corroborated by published work, albeit certainly not in an all-encompassing study. We chose to perform our demonstration with these known clinically used drugs so that the emphasis of our study can be the illustration of usability and the potential impact of our methodology rather than the electrophysiological insights *per se*.

## Discussion

The development of new pharmaceuticals, the comprehensive characterization and optimization of new human cell-based products, and the cardiotoxicity testing for all drugs require fast HT cellular interrogation. All-optical electrophysiology has the scalability potential and can provide a solution. Here we demonstrate how such an approach (OptoDyCE) can elevate cellular electrophysiology to the HT level. The ability for highly parallel dynamic stimulation is a key component of assessing arrhythmia propensity. While non-optogenetic (mostly thermally mediated) solutions are being pursued, opsin-based stimulation cannot be matched in terms of low energy, reliability and precision. We show computationally and experimentally that optogenetic stimulation is not interfering with the electrophysiological response of cardiomyocytes, and it can be realized in several simple ways. To our knowledge, the results here represent the first validation of the use of optogenetic actuators for drug-testing applications in cardiac electrophysiology. Particularly attractive for drug testing is the presented sOptoHTS method with sprinkled ‘spark' cells, which does not require any genetic modifications in the studied cells and can be easily incorporated in the current manufacturing workflow. Furthermore, our computational analysis illustrates that optogenetic sensors, which are often derived from ion-channel modules and other druggable components, may not be desirable in acute drug testing due to the temporal convolution of their response with the measured parameter of interest (AP or CT). While they are excellent reporters of activity long-term and *in vivo*, their use in the context of drug testing must be carefully validated. Instead, here we show that spectrally suitable synthetic optical sensors work well.

**Figure 1:**
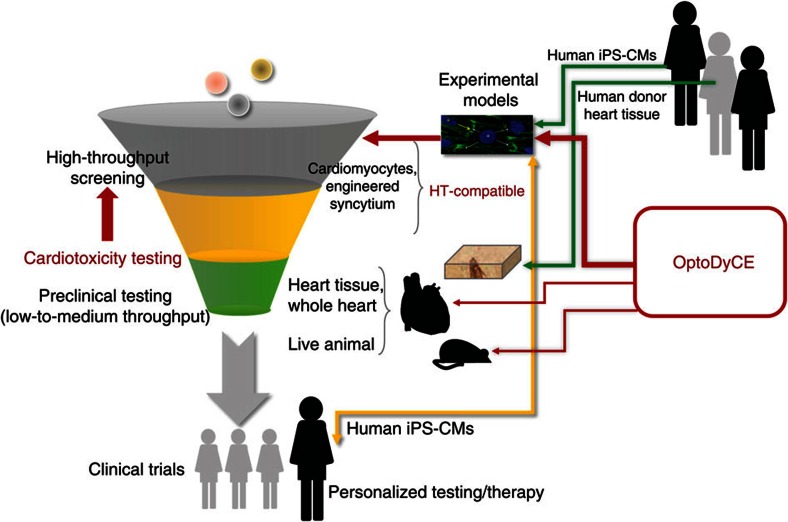
HT in drug discovery and cardiotoxicity testing. In the ‘funnel' workflow of drug development/drug testing, only some assays are HT-compatible. For cellular cardiac electrophysiology, currently there are no assays that fall into that category. The presented all-optical dynamic cardiac electrophysiology framework (OptoDyCE) is applicable to various experimental models (cells, tissue, whole heart and live animal testing) but only some of these (cardiomyocytes and some engineered syncytia, preferably using human iPS-CMs) are scalable and HT-compatible. Furthermore, they are the only ones that can be used directly for personalized testing/therapy on the same patient. Thick red arrows indicate that the OptoDyCE technology can elevate such functional cellular/multicellular cardiomyocyte assays for drug discovery or cardiotoxicity testing to HT-status. Further discussion of the best utility for different experimental models is provided in Supplementary Note 1.

The presented system, OptoDyCE, is the first scalable automated all-optical platform for cardiac electrophysiology that can meet the HT standard, Fig. 1. While often used colloquially in a liberal manner, ‘high-throughput' in the industrial setting of drug discovery and testing implies capability of performing over 10,000 assays a day (see considerations in Supplementary Fig. 5). HT requires that the samples and the process are scalable, manufacturing-friendly and amenable to handling with standard liquid- and cell-dispensing robotics within a standardized plate-format setting. With robotic dispensing of cells and drugs, the 96-well format, demonstrated here, can be instantly upgraded to 384-well or other standard plate formats, with very simple reprogramming. Thus, our system is indeed scalable. The current implementation has built-in parallelism within a well, interrogating hundreds of cells simultaneously (Supplementary Figs 5 and 6), but relies on serial traversing of the wells; a macroscopic version (see refs 24, 31) of OptoDyCE with larger field-of-view can further increase throughput by order(s) of magnitude. The all-optical approach can also be applied for the quantification of drug's action on cardiac conduction, including wave dynamics^24^, but with sacrifice of throughput due to space (spatial wavelength) required to accommodate such measurements.

The OptoDyCE framework is not limited to a particular experimental model. In addition to cultured cells, all-optical approaches can be used with cardiac tissue and whole hearts, including *in vivo* (Fig. 1). The contactless nature of interrogation in OptoDyCE makes it especially versatile and applicable to non-planar, 3D samples (Fig. 3), unlike any of the available technologies listed in Supplementary Table 1. As illustrated in Fig. 1, OptoDyCE can elevate electrophysiological testing to HT-status. This requires the experimental model to be HT-compatible, such as cardiomyocytes and small engineered tissues that can be cultured. Despite recognized current problems with human iPSC-CMs, namely immaturity and variability^32^, we argue that there may not be better alternatives when HT-format assays are considered. Native cardiac tissue (animal or human derived) provides a superior testing platform for assessing conduction abnormalities in a lower-throughput format (Fig. 1). However, it is not a suitable experimental target for the HT-level screening tests for several reasons: (a) there is limited availability of human heart tissue, and testing cannot be patient-specific because of the invasive nature of heart biopsies; (b) scalability is lacking due to spatial constraints—to make native tissue HT-compatible, the size of the individual tissue samples has to be reduced/cut markedly creating issues with handling; (c) viability and stability—cultured cell systems are inherently better suited for industrial scale handling and more stable than organotypic cultures. Furthermore, preclinical testing in live animals also plays a key role, as systems-level effects need to be probed. However, that is done in a low-throughput format at a later validating stage. More detailed discussion of the suitability of cardiac experimental models at different stages of drug testing is presented in the Supplementary Note.

In sum, HT electrophysiology with OptoDyCE is not intended to fully replace comprehensive *in vitro* (tissue or patch clamp) or *in vivo* tests, but it allows for quicker prediction of successful drug candidates, as well as more informed failure prediction to reduce cost in later testing phases. HT-compatible experimental models, such as based on iPSC-CMs, need to be further optimized to better mimic the native human heart properties, so that massively parallel interrogation enabled by OptoDyCE in the early phases of drug development can indeed make a difference in preventing drug failure at later stages in the process.

Our results also illustrate the extremely high-content data that can be obtained with the proposed platform. The ability to measure dynamically controlled APs, CTs and contractions and to extract a large number of arrhythmia-relevant parameters, including but not limited to frequency-dependent morphological changes (alternans, EADs, DADs, aftercontractions) as well as SCR, temporal and spatial variability of the response, allow the quantification of a drug's pro-arrhythmic risk in a much more comprehensive way than with any of the current platforms. Such comprehensive evaluation of the cellular responses is much better suited to capture the effects of more recently acknowledged non-classical multi-target modulators of ion channels, such as kinase inhibitors, for example^33^,^34^, compared with patch clamp data collection on a subset of individual ion channels, especially when done in non-myocytes. Drug effects mediated through intracellular-signalling pathways, for example, PI3K, may not be instantaneous and are likely to engage a large number of ion channels, in some of which the effects may be small or the measurements may be challenging (for example, measuring the late sodium current)^33^. Although not directly demonstrated here, such delayed/chronic action can be easily studied with the proposed platform, as cultured systems are stable over the relevant time frame (order of hours) and integral readouts (APs, CTs) are better and more relevant measures of such complex effects. A limitation of the optical interrogation methods, discussed here, compared with direct electrical measurements, is the difficulty in assessing absolute values, for example, resting membrane potential or diastolic calcium levels. Even with ratiometric measurements (using dual-wavelength sensors), calibration can be prohibitively difficult to apply in a HT setting. Nevertheless, drug-induced relative changes in these parameters can be inferred by the multitude of other measurements, as they directly influence the excitability and stability of the electrical response.

The high-content data present a challenge for automated processing, but also an opportunity that has not been available before. Data mining and analysis (for example, principal component analysis) of such massive number of outputs obtained in a self-consistent manner in the same experimental system under the same conditions, can provide a unique opportunity to design classifiers of a compound's cardiotoxity risk or of its ability to achieve desired safe modification of cardiomyocyte function. Such extensive high-quality data in response to a large number of known drugs is also invaluable as means to much needed tuning and constraining of currently pursued computer models for cardiotoxicity predictions at the cell and tissue level Supplementary Fig. 1. By offering a currently missing option for automated HT cardiomyocyte electrophysiology, OptoDyCE can also profoundly impact developments concerning human iPSC-CMs^10^,^11^ (see Supplementary Fig. 1) by allowing for combinatorial optimization of factors involved in cell maturation, phenotype selection and tissue engineering. In turn, the utilization of these new optimized human, potentially patient-specific, experimental models in conjunction with our HT testing platform has the potential to markedly improve preclinical drug testing, reduce cost, reduce animal use and increase a therapy's likelihood of clinical success.

## Methods

### Human iPS-Cardiomyocyte Culture and Gene Delivery

Frozen human iPSC-derived cardiomyocytes (iCell Cardiomyocytes^2^ CMC-100-012-001, Cellular Dynamics International (CDI), Madison, WI) were thawed per the manufacturer's instructions. The cells were plated at the recommended plating density of 156,000 cells cm^−2^ on fibronectin-coated 96-well glass-bottom plates. In some experiments, cells were grown in 3D microgrooved scaffolds. These fibronectin-coated PDMS (Sylgard 184, Dow Corning, Midland, MI, USA) scaffolds with microtopographical features (Fig. 3e, peak-to-peak 120 μm, depth of 50 μm) were produced by molding onto metal templates fabricated by acoustic micromachining^35^,^36^,^37^; and small circular microgrooved scaffolds were punched-out and placed in the 96-well plates. After 2 days, adenoviral delivery of *ChR2(H134R)* to the iPSC-CMs was performed in-dish, similar to that used for generation of primary rat ChR2-CMs (see details below). The maintenance medium was replaced with viral doses of multiplicity of infection (MOI) 10, 15, 50, 100, 250, 500 and 1,000 prepared in Opti-MEM (GIBCO). Cells were incubated (37 °C, 5% CO_2_) and gently agitated every 20 min for a total of 2 h, after which cells were returned to the maintenance media. Functional and structural testing was then performed 2 days after viral delivery to determine the optimal MOI.

### Primary Cardiomyocyte Isolation and Culture

Primary cardiomyocytes (CMs) used through the study for all illustrative experiments with pharmacological treatments, mainly because at the current stage, they are perceived as more mature than the hiPSC-CMs and their response to drugs has been better documented. Briefly, neonatal (2–3-day old) Sprague–Dawley rats were killed and ventricular tissue was removed per an approved Stony Brook University IACUC protocol. The tissue was digested overnight at 4 °C using 1 mg ml^−1^ trypsin (US Biochemicals, Cleveland, OH) in Hanks' Balanced Salt Solution (HBSS, GIBCO Invitrogen, Carlsbad, CA). The next morning, the tissue was serially digested using 1 mg ml^−1^ collagenase (Worthington Biomedical, Lakewood, NJ) in HBSS at 37 °C and pipetted into conical tubes and placed on ice. After centrifugation, cells were re-suspended in culture medium M199 (GIBCO) supplemented with 12 μM L-glutamine (GIBCO), 0.05 μg ml^−1^ penicillin-streptomycin (Mediatech Cellgro, Kansas City, MO), 0.2 μg ml^−1^ vitamin B12 (Sigma-Aldrich, St. Louis, MO), 10 mM HEPES (GIBCO), 3.5 mg ml^−1^ D-(+)-glucose (Sigma-Aldrich) and 10% fetal bovine serum, FBS (GIBCO). Fibroblasts were removed via a two-step pre-plating process, where the cell suspension was plated in a flask and incubated (37 °C, 5% CO_2_) for 45–60 min and switched to a new flask and the incubation repeated. CMs were then counted using a hemocytometer before plating in glass-bottom 96-well plates^27^,^37^.

### Gene and Cell Delivery for Optogenetic Actuation

Introduction of the optogenetic actuator was performed via direct gene delivery into cardiomyocytes using an adenovirus (ChR2-CM)^25^,^26^ or via ‘spark' cells (here an in-house developed stable ChR2-HEK293 cell line was used; other light-sensitized somatic cells can be used as well), based on a variation of the ‘tandem-cell-unit' concept^27^. Through the text, for the general HT implementation of OptoDyCE, especially involving directly infected ChR2-CMs, we use OptoHTS, while for the specific implementation with ‘spark' cells we use sOptoHTS.

Adenoviral delivery of *ChR2(H134R)* to primary cardiomyocytes was performed in suspension^26^. Briefly, we used the plasmid *pcDNA3.1/hChR2(H134R)-EYFP* from Addgene (Cambridge, MA), deposited by Dr. K. Deisseroth^38^, to develop an adenoviral construct (*pBR322* backbone) with a ubiquitous CMV promoter. First-generation adenovirus was generated by homologous recombination of the *Ad-CMV-ChR2-eYFP* into *pTG3604*; further propagation and purification of the virus genomes was done by transfection into HEK293 cells and CsC1 banding. CMs were re-suspended in 2% FBS M199 after counting and diluted to 1.125 × 10^6^ cells ml^−1^ and infected using an optimized MOI of 15 for 2 h in an incubator (37 °C, 5% CO_2_) with gentle agitation every 20 min. The MOI had been optimized during preliminary experiments to achieve >95% ChR2 expression in CMs within 48 h using titre of 10^12^ units ml^−1^ diluted in PBS, confirmed by eYFP reporter visualization and minimal cell death (by propidium iodide staining)^15^,^26^. After 2 h, the cell suspension was centrifuged and culture medium was removed and replaced with fresh 10% FBS M199 for plating^26^.

The cell delivery approach use ‘spark' cells (*ChR2(H134R)-HEK293*), a stable cell line showing near 100% expression of ChR2. ‘Spark' cells were developed by transfecting HEK293 cells (CRL-1573 ATCC, Manassas, VA) with the ChR2 plasmid *pcDNA3.1/hChR2(H134R)-EYFP* using Lipofectamine 2000 (Invitrogen, Carlsbad, CA). ChR2-expressing cells were then selected by the application of 500 μg ml^−1^ geneticin (Invitrogen)^27^. Before use, the ChR2-HEK cells were grown at 37 °C , 5% CO_2_ in DMEM (GIBCO Invitrogen) supplemented with 10% FBS and 1% penicillin-streptomycin. After trypsinization, the cells were delivered by one of two methods during plating: co-culture, where the cells were mixed with CMs at the time of plating, or ‘sprinkling' of ‘spark' cells on top of already plated cardiomyocytes 24–48 h before experiments.

### Cell Plating

For all cell conditions, 50 μg ml^−1^ fibronectin, diluted in PBS, was used on 96-well glass-bottom plates (*In Vitro* Scientific) and incubated at 37 °C for at least 2 h before cell plating. Cells were plated in 10% FBS M199 media; on day 2, the media was replaced with 2% FBS M199 until the day of experiments.

Non-infected control CMs and ChR2-CM cells (for OptoHTS) were plated using a concentration of 1.125 × 10^6^ cells ml^−1^ to achieve a plating density of 470,000 cells cm^−2^. For CM/ChR2-HEK co-cultures, CMs at a concentration of 1.125 × 10^6^ cells ml^−1^ were mixed in a conical with trypsinized ChR2-HEK cells, pre-diluted to achieve ratios 150:1 CM to ChR2-HEK. After centrifugation, the media was removed and the cells were re-suspended in fresh 10% FBS M199 to achieve the same density of plating as the control cardiomyocytes.

ChR2-HEK cell ‘sprinkling' (for sOptoHTS) was performed by exchanging culture media of already plated CMs with a cell suspension of ‘spark' ChR2-HEK cells, diluted to achieve ratios of 75:1 CM to ChR2-HEK cells. In this approach, the CMs were diluted to either 1.075 × 10^6^ cells ml^−1^ and cultured for several days. 48 h before experiments, ChR2-HEK cells were trypsinized and diluted in 2% FBS M199 to achieve the desired plating ratios at the required volume for a media exchange. Media in the dishes containing CMs was then removed and replaced with the ChR2-HEK cell suspension.

### Automated All-Optical Electrophysiology

All functional experiments were carried out 4–5 days after cell plating, at room temperature in Tyrode's solution containing the following (in mM): NaCl, 135; MgCl2, 1; KCl, 5.4; CaCl2, 1.5; NaH2Po4, 0.33; glucose, 5; and HEPES, 5 adjusted to pH 7.4 with NaOH^37^. The optical setup (Fig. 3o,p) was built around an inverted microscope (Nikon Eclipse TE-2000-U) fitted with a programmable *x*–*y* stage (OptiScan ES107; Prior Scientific; Rockland, MA) and automated *z*-focus (PS3H122 Motorized Focus; Prior Scientific). Illumination for optical actuation and sensing was provided by TTL-programmable LEDs coupled into the system using a custom-built adaptor. Optical actuation of ChR2 was provided by aLED, a 470 nm, 650 mW LED (Thorlabs; Newton, NJ), controlled by an LED driver (Thorlabs), and fitted with a 470/28 nm bandpass filter, F_actu_.

The components of the optical sensing light-path were selected based on the optical sensor. Voltage measurements, *V*_m_, were recorded using the synthetic voltage-sensitive dye Di-4-ANBDQBS^39^ (from Leslie Loew, University of Connecticut) with fluorescence excitation and emission peaks at 660 nm and >700 nm, respectively. Rhod-4AM (AAT Bioquest, Sunnyvale, CA) with fluorescence excitation and emission peaks at 530 nm and 605 nm, respectively, was used for intracellular calcium, [Ca^2+^]_i_ recording.

Illumination for sensing was provided by sLED (V_m_: 640 mW LED at 660nm; or [Ca^2+^]_i_: 350 mW LED at 530 nm, both from Thorlabs), fitted with a bandpass filter F_ex_ (V_m_: 655/40 nm; or [Ca^2+^]_i_: 535/50 nm). The light paths for optical sensing and actuation were combined by a dichroic mirror DM1 (495 nm long-pass) and directed to the sample by DM2 (V_m_: 685 nm long-pass; [Ca^2+^]_i_: 565 nm long-pass). Collimation optics comprised of several lenses (L), and an objective lens (in this case 20 × Nikon CFI Super Plan Fluor) was used to direct light to the sample. Emitted fluorescence was collected by the objective lens and passed through DM2 and a bandpass emission filter F_em_ (*V*_m_: 700 nm long-pass; [Ca^2+^]_i_: 605/70 nm bandpass) to a photodetector (in this case, iXon Ultra 897 EMCCD; Andor Technology Ltd., Belfast, UK).

The main experiments were carried out using 96-well plates, automated as shown in Fig. 3i,j. The stage was programmed to traverse each well (*x*–*y* coordinates), auto-focus (*z* coordinate) on the sample under constant fluorescent illumination and then record for 5–20 s, based on the desired optical pacing protocol. The optical pacing LED was controlled via TTL to deliver the desired pacing protocol, and the optical sensing LED was constantly on, while the camera was programmed to record only during the pacing. This protocol was repeated for each well over the entire dish. If needed, fluorescent images of the dye and of eYFP of the actuator were also recorded using an automated protocol. After recording, intensity values over time averaged over the full fields of view (FOVs) or extracted per region were exported for post-processing.

### Code availability

The settings and software for automation, post-processing and data analysis is available for download in the Supplementary Information and at http://entcheva.seas.gwu.edu/software.

### Optical Pacing and Optical Recording

Optical recording of membrane voltage, *V*_m_, was performed using the synthetic voltage-sensitive dye Di-4-ANBDQBS, spectrally compatible with ChR2. Briefly, a 17.5 mM stock solution in pure ethanol was diluted to 35 μM Tyrode's solution. Cells were stained for 6 min in dye solution followed by a 6 min wash in fresh Tyrode's. This wash solution was then replaced by fresh Tyrode's. Intracellular calcium, [Ca^2+^]_i_, was recorded using Quest Rhod4 AM diluted from a 0.5 mM stock solution dissolved in DMSO with 20% Pluronic to 10 μM in Tyrode's solution. Samples were stained with this solution for 20 min, followed by a 20 min wash in fresh Tyrode's solution, and finally a replacement with fresh Tyrode's before experiments.

Optical imaging was performed at >200 frames per second (fps) with 4 × 4 binning using NIS-Elements AR (Nikon Instruments; Melville, NY). Optical stimulation (470 nm) was provided at pulse lengths of 5–20 ms, at 0.5–8 Hz, using irradiances of 0.4–7 mW mm^−2^, as needed. Electrical stimulation (for comparing electrical versus optical stimulation) was delivered through a pair of parallel platinum electrodes connected to a pulse generator (IonOptix, Milton, MA) providing 5 ms 10 V bipolar pulses at 0.5–2 Hz.

Comparison of electrical pacing of control CMs (without gene or cell delivery of ChR2) with optical and electrical pacing of ChR2-CM and CM/ChR2-HEK cells was carried out on 14-mm glass-bottom dishes. Multiple FOVs were taken per dish, and each recording was divided into smaller region of interest (ROI) and intensity data over time was extracted and analysed. Contractions were measured by post-processing of recorded videos and tracking cell motion (displacement) by naturally occurring cell heterogeneities (fiducial markers).

### Immunocytochemistry

To confirm ChR2 expression (in the primary rat CMs, in the CM/ChR2-HEK co-cultures and the iPS-CMs) and to confirm myocyte-like properties of iPS-CMs, antibody staining and confocal imaging was performed (Fig. 3a,c and Supplementary Fig. 2), using the Olympus FluoView FV1000 confocal system. Samples were fixed in 3.7% formaldehyde after performing functional experiments. Before antibody labelling, cell membrane permealization was performed by incubating samples in 0.02% TritonX-100 for 5 min. Cells were labelled with mouse anti-α-actinin primary antibody (Sigma-Aldrich, A-7811) at 1:600 and Alexa Fluor 647 goat anti-mouse IgG secondary antibody (Invitrogen, A21235) at 1:1,000. All antibodies were diluted using 1% bovine serum albumin (Amersham PLC, Amersham, UK). 1% FBS was used as a blocking agent. After antibody staining, cell nuclei were stained with 1 μg ml^−1^ DAPI with 10 min incubation in PBS. Imaging was done using the Olympus FluoView FV1000 confocal system with acquisition rate at 4 μs per pixel. Gain was kept constant for control and test groups to normalize and exclude autofluorescence contributions.

### Drugs

Nifedipine (MW 346.33 g mol^−1^; Sigma) concentrations of 50 μM, 10 μM, 5 μM, 1μM, 0.5 μM, 0.1 μM, 0.05 μM, 0.01 μM, 0.005 μM, 0.001 μM and 0.0001 μM were prepared in Tyrode's solution. A stock solution of 100 mM in DMSO was serially diluted in DMSO and then Tyrode's solution to the final concentrations. Dofetilide (MW 441.56 g mol^−1^; Fisher) concentrations of 2 μM, 0.2 μM, and 0.02 μM were prepared in Tyrode's solution. A stock solution of 200 mM in DMSO was prepared and serially diluted in DMSO and then Tyrode's solution to the final concentrations. DMSO was not seen to have effect on APD at the used concentrations (<1% DMSO; Supplementary Fig. 10). Drug doses were dispensed manually after staining and washing and before experiments. Measurements were completed within 20–50 min after drug application, assuming steady-state of action.

### Data Processing and Analysis

Data was analysed using custom-developed HT software in MATLAB, flashligHTS (Supplementary Fig. 8). The software was used to automatically extract ‘events' from recorded traces, that is, AP and CTs, as well as to quantify certain morphological features of these events, while keeping track of wells (samples) and spatial locations (or ROIs). Data pre-processing included baseline correction, removal of artefacts, temporal filtering using a Savitzky-Golay polynomial filter (second order, 7–11 frame window) and normalization. All traces are reported in terms of per cent change of fluorescence from baseline (ΔF/F) and normalized per cent ΔF/F. All normalized example APs and CTs were averaged over 6–10 beats of filtered traces. APDs and CTDs at 25, 50 and 80% were automatically extracted and defined as the time difference between the onset of an AP/CT and the point of 25, 50 and 80%, respectively, return to baseline. Contractions were quantified by video post-processing and expressed as relative displacement (in per cent length change). Phase maps to capture wavefront irregularities and localized abnormal activations (as shown in Supplementary Fig. 6) were constructed using the Hilbert transform, as described in our earlier work^40^.

Data are presented in terms of mean±standard error of the mean (s.e.m.). For drug-dosing experiments, APD/CTD data for each group are calculated by finding the average APD/CTD for each well (usually 6–10 beats). All averaged APD/CTDs for each well for a condition (for example, drug dose) are then averaged and the s.e.m. is calculated. ΔAPD/CTD (%) is given as the per cent change of this calculated average from the average of the control group (for example, no drug). s.e.m. is calculated by appropriately propagating the s.e.m. from each group. Data combined over different cultures is only given in terms of ΔAPD/CTD (%). Here, for each run, the ΔAPD/CTD (%) is calculated with respect to the average control APD/CTD (over all control wells). These normalized ΔAPD/CTD (%) for each well are then combined across all runs and averaged.

Variations within a sample (temporal variation of events or spatial variation of events within the FOV) are quantified using the MAD as a measure of variability. MAD is calculated by taking the median of the absolute deviations from the data's median (Supplementary Equation 1) for APD25/50/80 or CTD25/50/80 for each sample and then averaged across samples.


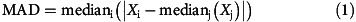


Statistical comparison of OptoHTS and sOptoHTS APDs was performed in MATLAB using an ANOVA test followed by a Tukey–Kramer *post hoc* correction for multiple comparisons. Values of *P*<0.01 were considered statistically significant.

### Computational Analysis of Optogenetic Sensors and Actuators

**Figure 2:**
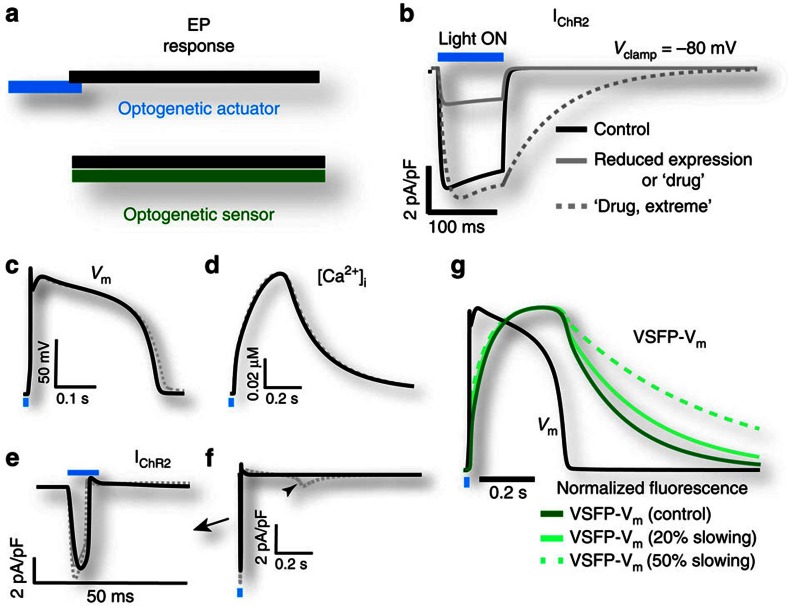
Drug testing considerations and computational analysis of the use of optogenetic tools. A comparison of actuators (for example, ChR2) versus optogenetic sensors (for example, VSFP2.3) in human ventricular myocytes. A fast optogenetic actuator acts as a stimulation impulse and is ‘time-detached' from the electrophysiological response (**a**), and therefore a hypothetical drug action that affects ChR2 current amplitude and/or kinetics (**b**) has minimal effect on the optically triggered APs (**c**) and CTs (**d**), if light irradiances are adjusted to provide supra-threshold currents (**e**). Even extreme drug interference with ChR2 off-kinetics results in minor (5%) APD prolongation (**c**) due to re-activation of inward ChR2 current during repolarization (**f**). In contrast, an optogenetic sensor, for example, VSFP2.3, is fully ‘temporally convolved' with the electrophysiological response (**a**), and even a mild drug action on the sensor can profoundly influence the electrophysiological readout (**g**).

**Figure 3:**
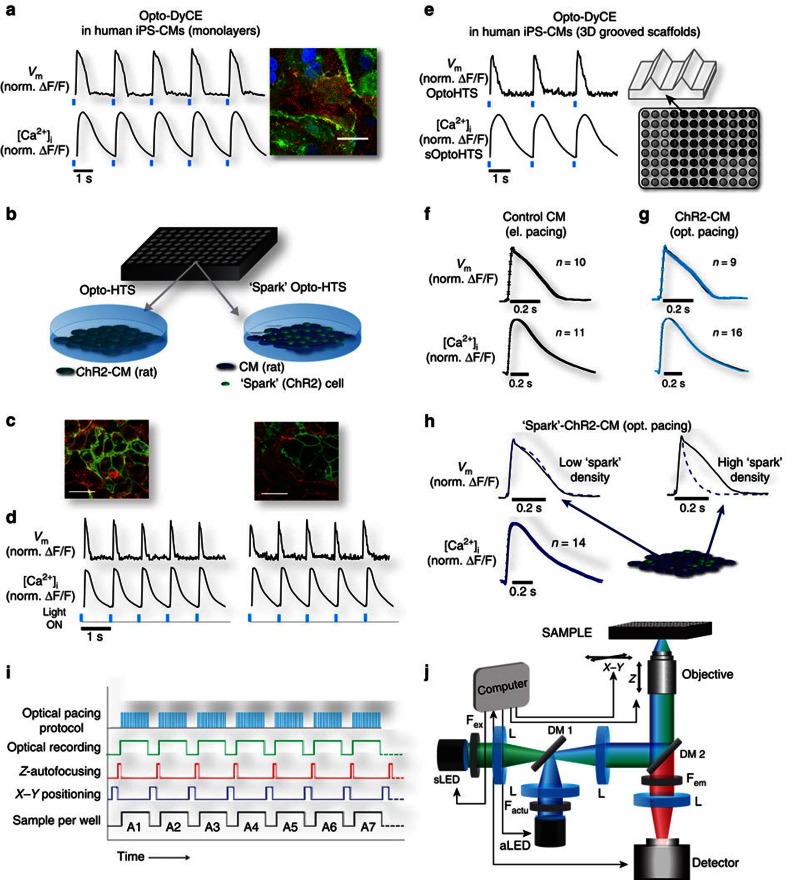
Experimental HT implementation and validation of automated OptoDyCE. Human ChR2-iPSC-CMs in a monolayer (**a**) or in 3D structures (**e**), and rat ChR2-CMs (OptoHTS) and ‘spark'-ChR2-CMs (sOptoHTS) (**b**,**c**,**d**,**g,h**) are optogenetically transformed to respond to optical stimulation. ChR2 expression by eYFP reporter (green), α-actinin staining (red) and DAPI nuclear stain (blue) are shown (**a**,**c**). Scale bar, 30 μm, 25 μm (**a** and **c**, respectively). Optical pacing reliably triggers *V*_m_ and [Ca^2+^]_i_ signals, measured optically (**a**,**d**,**e**). Validation of OptoHTS comes from identical AP and CT morphology for electrically paced CM-controls (non-transduced) and optically paced ChR2-CMs (**f**,**g**); in sOptoHTS, high ‘spark' cell density can lead to APD shortening compared with control CM (**h**) (Supplementary Fig. 4) without much effect on CT morphology (**h**). We demonstrate a fully automated HT version of OptoDyCE in 96-well format (**b**) using a custom-built optical setup and an automation protocol (**i**,**j**) (for details see the subsection ‘Automated All-Optical Electrophysiology' in the ‘Methods' section).

**Figure 4:**
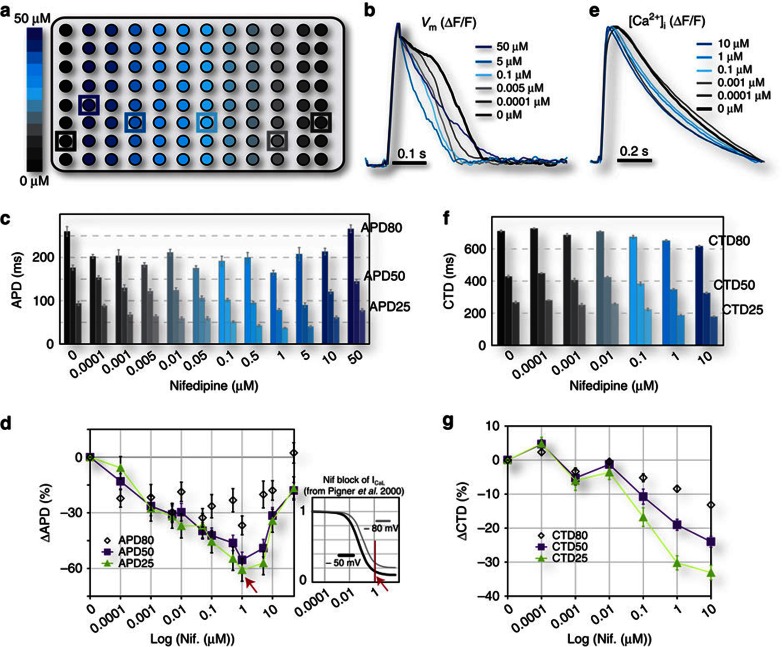
Demonstration of OptoHTS for HT dose–response drug testing. Nifedipine, an L-type Ca^2+^ channel (*I*_CaL_) blocker, is applied at 12-concentration-graded dosing (0–50 μM) to ChR2-CMs in 96-well plates (**a**). Optical recordings of multiple voltage (**b**–**d**) or calcium events (**e**–**g**) are obtained during optical pacing at 1 Hz, screening the full plate in under 10 min (see also Supplementary Fig. 5). Example averaged over 10 s (**b**,**e**) and quantitative results for APD and CTD (**c**,**d**,**f**,**g**) are shown. Expected APD (*n*=4–7 samples, at least 800 single-cell records per concentration) (**b**–**d**) and CTD (*n*=4–6 samples, at least 800 single-cell records per concentration) (**e**–**g**) shortening, especially at the APD25/CTD25 and APD50/CTD50 levels, occurred due to nifedipine blocking the inward L-type calcium current. Maximum APD shortening is observed at around 1 μM, consistent with maximum block of *I*_CaL_ reached at that concentration (**d**, inset). Beyond 1 μM, indirect (voltage-mediated) or non-specific action on other ion channels partially counters the block of inward Ca^2+^ current and can reduce or eliminate the APD shortening (**d**). Nifedipine appears to monotonically shorten CTD up to 10 μM (**f**,**g**). Data are presented as mean±s.e.m, and each well is considered an independent sample, represented by a spatially averaged trace.

**Figure 5:**
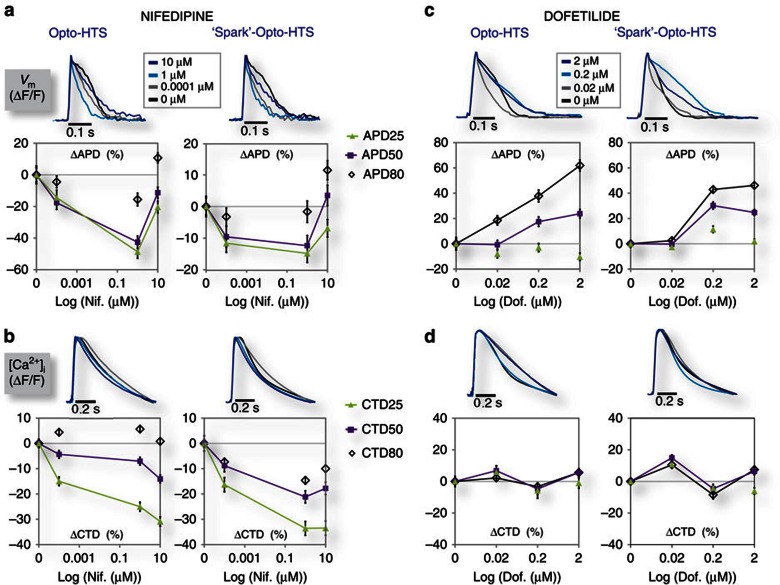
Experimental comparison of OptoHTS versus ‘spark'-OptoHTS for functional drug testing. (**a**–**d**) OptoHTS (left) and sOptoHTS (right) provide qualitatively and quantitatively similar results for measured effects on APD (**a**,**c**) and CTD (**b**,**d**) for both nifedipine (**a**,**b**) and dofetilide, a blocker of the rapid delayed-rectifier, *I*_Kr_ (**c**,**d**). *N*=3–16 samples (at least 600 single-cell records or more) for each condition and each data point in the panels above. Data are presented as mean±s.e.m., and each well is considered an independent sample.

**Figure 6:**
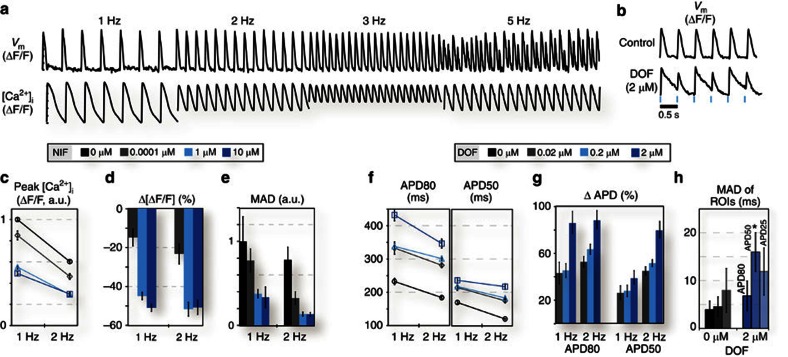
Dynamic functional drug testing. A demonstration of the utility of OptoDyCE for spatio-temporal characterization. Dynamic pacing provides a means of studying pacing-induced *V*_m_ and Ca^2+^ restitution and instabilities (**a**), or drug-induced instabilities, that is, 2 μM dofetilide leading to voltage alternans at relatively low pacing frequency (2 Hz) (**b**). High-content dynamic information is obtained from a single data run (**c**–**h**). For example, restitution and temporal or spatial variability (quantified by MAD) are shown as function of both drug dose and pacing frequency for peak calcium in the presence of nifedipine (**c**–**e**) and for APD in the presence of dofetilide (**f**,**g**). Nifedipine action on peak calcium (per cent change) is dose-dependent but frequency-independent (**c**,**d**). Nifedipine appears to reduce temporal variability of peak calcium (assessed by MAD), and this reduction is augmented by higher frequency pacing (**e**). Dofetilide shows enhanced action on APD50 at higher frequency (opposite to reverse-use dependence) (**f**,**g**). Spatial variation as a function of drug dose can also be assessed by analysing multiple ROI within the same well (**h**) (see also Supplementary Figs 5–6). Dofetilide at 2 μM seems to increase spatial variability in APD, that is, increase dispersion of repolarization, compared with control during 1-Hz pacing (*P*<0.05 for APD50 obtained using ANOVA test followed by a Tukey–Kramer *post hoc* correction for multiple comparisons). *N*=5–16 samples (at least 1,000 single-cell records or more) for each condition and each data point in the panels above. Data are presented as mean±s.e.m., and each well is considered an independent sample.

Computational modelling of *ChR2(H134R)* action in human ventricular myocytes^41^, shown in Fig. 2b–g and Supplementary Fig. 4a, was performed in MATLAB. Briefly, a four-state model of ChR2 with nonlinear voltage and light dependence was integrated into a human ventricular cardiac model^25^,^28^. To simulate a reduced expression or purported drug inhibitory action, we reduced the ChR2 conductance by 70%; while ‘drug, extreme' action was modelled by 70% reduced conductance and 95% slowing of G_**d1**_ and G_**d2**_ transition rates in the ChR2-state model (Fig. 2b–f). Irradiance was adjusted/increased to trigger a response in Fig. 2c–f under the abnormal conditions. The action of the optogenetic voltage sensor VSFP2.3 in human ventricular myocytes was simulated using a state model of VSFP2.3 (ref. 23). In Fig. 2g, VSFP-V_m_ reports the fluorescence measured in a human ventricular myocyte. The ‘20% slowing' case was simulated by using a 0.8 scaling of all OFF rate constants in the model, and the ‘50% slowing' case was simulated by using a 0.5 scaling factor of all OFF rate constants^23^.

To simulate conditions in the ‘spark' OptoHTS approach, we used a human ventricular myocyte model^41^, coupled to fibroblast(s)^42^, modified to express ChR2 (ref. 25). Multiple light-sensitive fibroblasts could be coupled to a cardiomyocyte to capture the electronic effects they have on APD, as shown in Supplementary Fig. 4a. The effects of HEK cells would be qualitatively similar to the modelled fibroblasts, in the sense that they are both electrically passive cells with more depolarized resting membrane potential than the cardiomyocytes. Software for the described computer simulations is available for download in the Supplementary Information and at http://entcheva.seas.gwu.edu/software.

## Additional information

**How to cite this article**: Klimas, A. *et al*. OptoDyCE as an Automated System for High-Throughput All-Optical Dynamic Cardiac Electrophysiology. *Nat. Commun.* 7:11542 doi: 10.1038/ncomms11542 (2016).

## Supplementary Material

Supplementary Information Supplementary Figures 1-10, Supplementary Table 1, Supplementary Note 1 and Supplementary References

Supplementary Software 1 — FlashLigHTS: automated software for transient analysis. (See Supplementary Figure 8: flashligHTS analysis software.) 2 — NIS_Automation_Template.xml: template for Nikon NIS software for high-throughput protocols 3 — Coupled Cardiomyocyte – Spark Cells Model: code for modeling the effect of "spark" cell density on measured APDs in CMs. (See Supplementary Figure 4: Effect of "spark" cell density on measured APD in CMs.) 4 — Model of Cardiomyocyte with Optogenetic Sensor and Actuator: code for modeling the effect of drug action on an optogenetic sensor versus optogenetic actuator (See Figure 2 Drug testing considerations and computational analysis of the use of optogenetic tools.)
