# Supplementary material for: OptoDyCE as an automated system for high-throughput all-optical dynamic cardiac electrophysiology
Source: Nat Commun. 2016 May 10;7:11542. doi: 10.1038/ncomms11542 (PMC4866323; doi:10.1038/ncomms11542)
Supplement: Supplementary Information — Supplementary Figures 1-10, Supplementary Table 1, Supplementary Note 1 and Supplementary References [file ncomms11542-s1.pdf]

Supplementary Information

Supplementary Figures

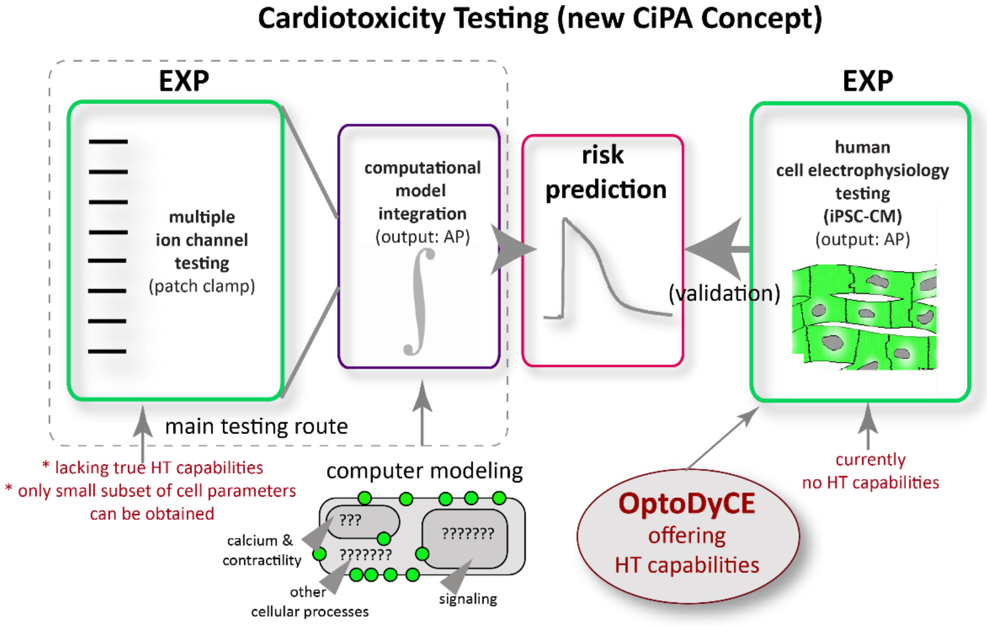

Supplementary Figure 1: The new CiPA concept and a role for OptoDyCE in cardiotoxicity testing. The new CiPA[1] (Comprehensive In Vitro Pro-arrhythmia Assay) concept aims to help change/improve current ICH* regulations for pre-clinical cardiotoxicity testing to avoid unnecessary drug attrition, reduce cost, and improve drug development. The arrhythmia risk is to be derived from experimental data using multi-channel testing by manual or planar automated patch clamp in recombinant expression systems (see IonWorks in **Supplementary Table 1**), which are then integrated using computational tools to predict the overall action of a compound on human cell electrophysiology, i.e. on the action potential (AP) - this experimental step plus the computational step form the main testing route. The predictions (the effects of a compound on the human action potential) are to be validated using cell electrophysiology measurements in human cardiomyocytes (most likely, iPSC-CMs). Note that currently, both of the two experimental components in this scheme lack true high-throughput (HT) capabilities. Planar patch systems have evolved but do not pass the HT threshold; cardiomyocyte electrophysiology (AP measurements) currently cannot be performed in HT fashion. Our platform, OptoDyCE, aims to bring HT capabilities to the cell-level testing in human cardiomyocytes. This experimental approach (on the right) is more direct and can theoretically (pending maturation of the iPSC-CM technology) provide more relevant, and even patient-specific predictions, compared to the main testing route on the left (a comprehensive characterization of all ion channels, signaling and other intracellular processes is impossible, and hence the complex computational models operate in high level of uncertainty, thus providing only probabilistic predictions). Furthermore, OptoDyCE can provide additional cell- and multicellular readouts, e.g. intracellular calcium, contractility, cell coupling, which are very relevant to arrhythmia testing but cannot be derived by the approach on the left. Therefore, OptoDyCE can help further constrain/improve computational modeling as well.

*ICH - International Conference on Harmonization of Technical Requirements for Registration of Pharmaceuticals for Human Use – coordinated regulatory efforts by Europe, Japan and the United States concerning pharmaceutical products.

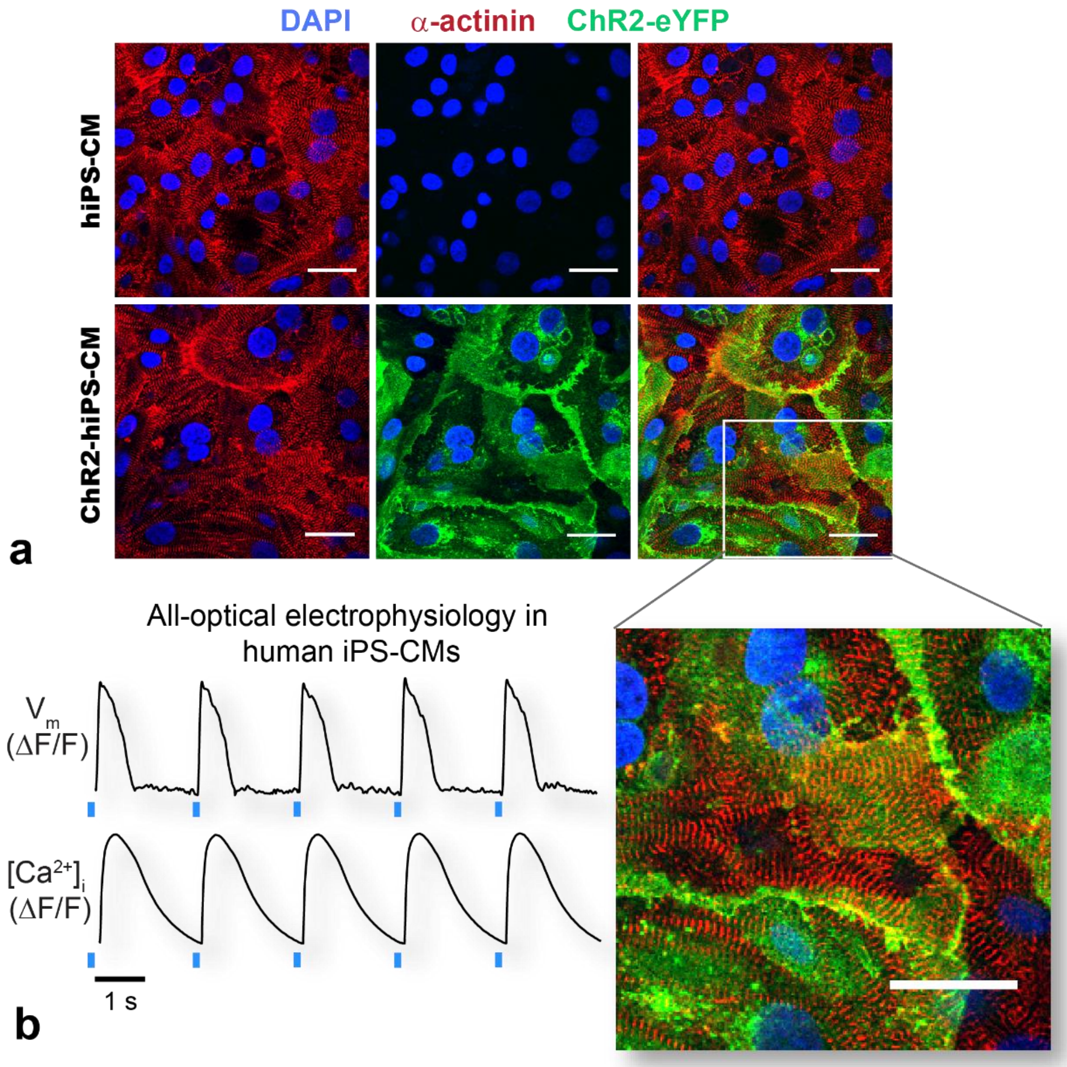

Supplementary Figure 2: All-optical electrophysiology in human iPS-CMs (expanded Fig. 2a). (a) hiPS-CMs without (top) and with Ad-ChR2(H134R)-eYFP delivery at MOI 250 (bottom). Red fluorescence indicates α-actinin staining illustrating the cardiomyocyte-like properties of hiPS-CMs, blue indicates DAPI nuclear staining, and green fluorescence indicates the eYFP reporter of ChR2. Combination (left) of the α-actinin (right) and eYFP (center) channels indicate expression of ChR2 in the ChR2-hiPS-CMs. Scale bar is 30 μm. **(b)** Optical recording of V_m and $[Ca^{2+}]_i$ in optically paced ChR2-hiPS-CMs, used in an automated readout (96-well plate format).

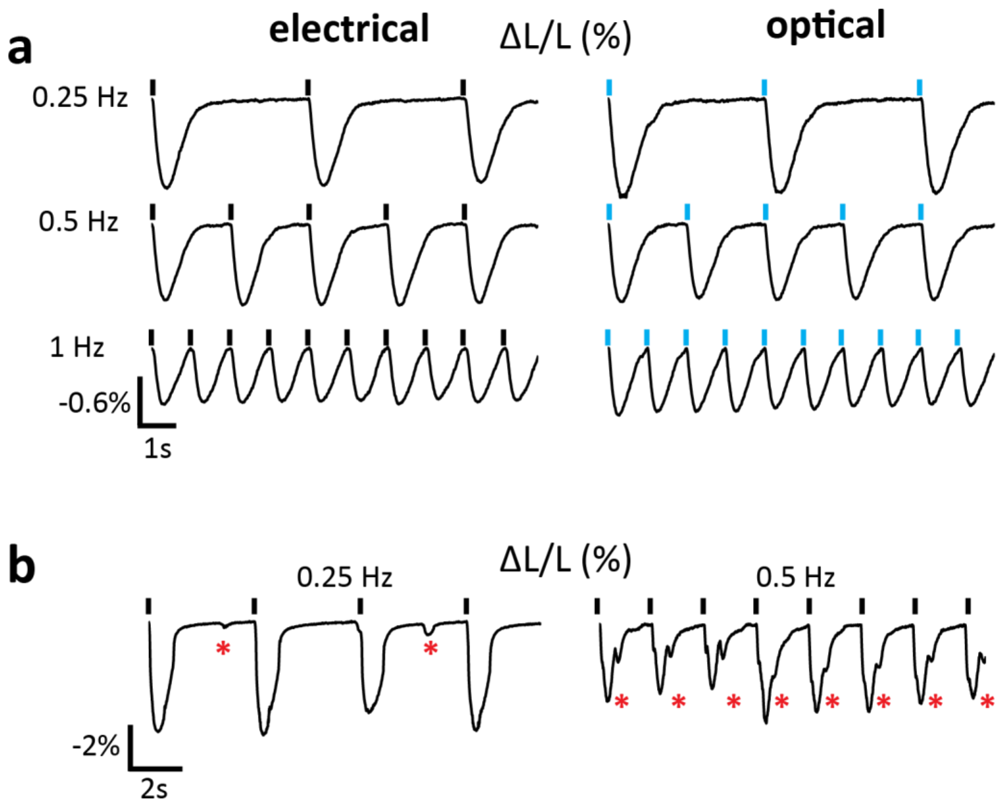

Supplementary Figure 3: Capturing cardiomyocyte contractions with OptoDyCE. (a) Validation of comparable contractility in response to optical (90 ms, 0.015 mW/mm^2) vs. electrical (5 ms, 10 V bipolar) stimulation under different pacing frequencies; tick marks indicate stimulation pulses. Shown are measurements in neonatal rat ventricular ChR2-CMs. **(b)** The OptoDyCE system is capable of resolving abnormal contractile responses, e.g. aftercontractions (red asterisks) that can be used as arrhythmogenic markers.

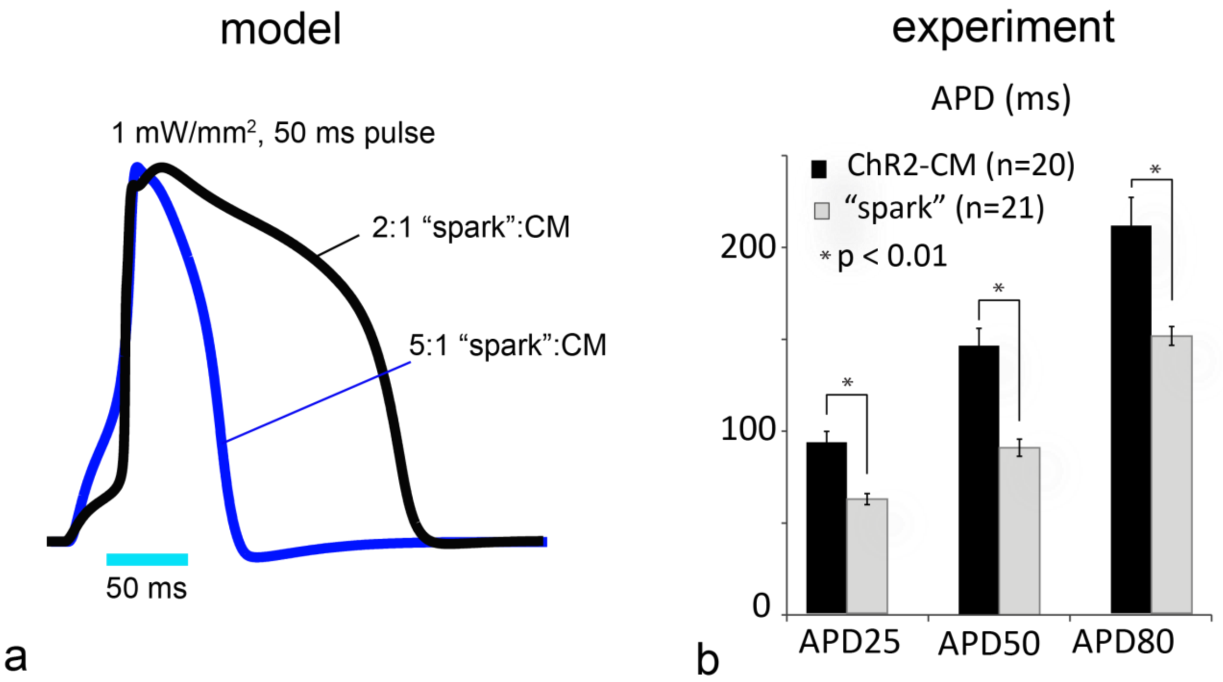

Supplementary Figure 4: Effect of "spark" cell density on measured APD in CMs. (a) Shown is a computational example of human ventricular action potentials, scaled and normalized in amplitude (as would be measured by an optical method) for two cases of "spark"-driven excitation of CMs: when 2 "spark" cells were connected to a cardiomyocyte or when 5 "spark" cells were connected to a cardiomyocyte. The loading effect in the latter case resulted in APD shortening in the CM (see **Fig. 2h**). The computer model employed ChR2-expressing cardiac fibroblasts (not HEK cells) as "spark" cells (see *Computational Analysis*), but the effect is applicable to both cell types. **(b)** Comparison of the experimentally measured APD for optically paced ChR2-CMs and HEK-ChR2-CMs for the samples shown in **Fig. 2g-h**. For the cell density and the implementation here, with random sprinkling, there was overall APD shortening in the "spark"-driven (HEK-ChR2-CMs) compared to the ChR2-CMs ($p < 0.01$ using ANOVA test followed by a Tukey-Kramer post hoc correction for multiple comparisons.); to avoid APD shortening, the "spark" cells can easily be localized and can serve as optical pacemaking conduits without affecting the APD of the cardiomyocytes.

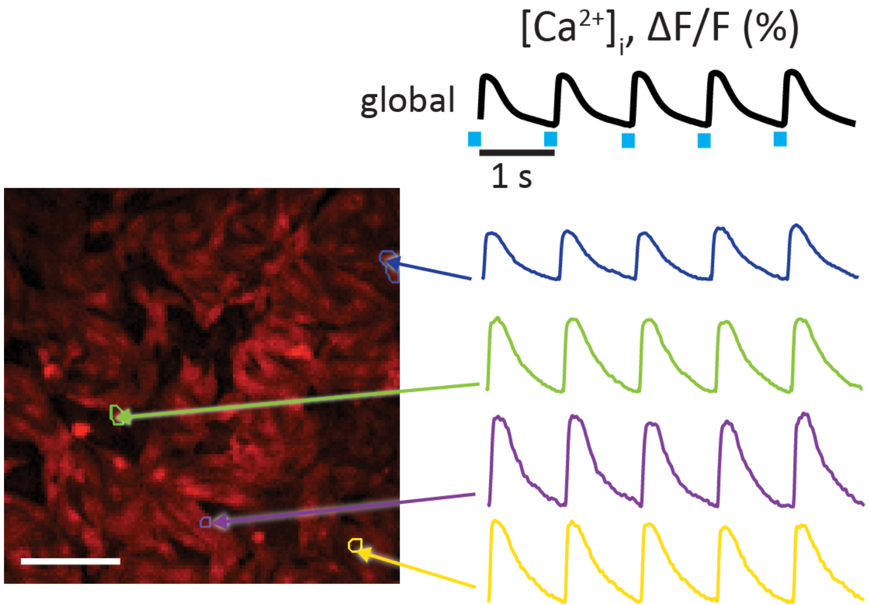

Supplementary Figure 5: Parallelism of the OptoHTS system. In each well, optical recording was performed over multiple cells in parallel. Typical field of view (FOV) was 400 μm x 400 μm, resulting in about 200-400 parallel cell measurements per FOV (well), i.e. about **30,000 cell-level measurements per 96-well plate** at 20x magnification. For a dynamic pacing protocol, using **multi-beat pacing** (6 second dwell time per well), this resulted in about **10 min/plate**, i.e. about **600 independent multi-cellular (>200 cells) samples (or compounds) per hour** (with the possibility to reach **> 10,000 compounds per day**, which qualifies for HTS). Shown here are the global (space-integrated) calcium measurement for a well (scale bar 100 μm) and traces from individual cell-level regions, as outlined. While most of the analysis presented here dealt with the global responses, the parallelism is built-in into our approach and can easily be utilized further (see **Fig. 5h**).

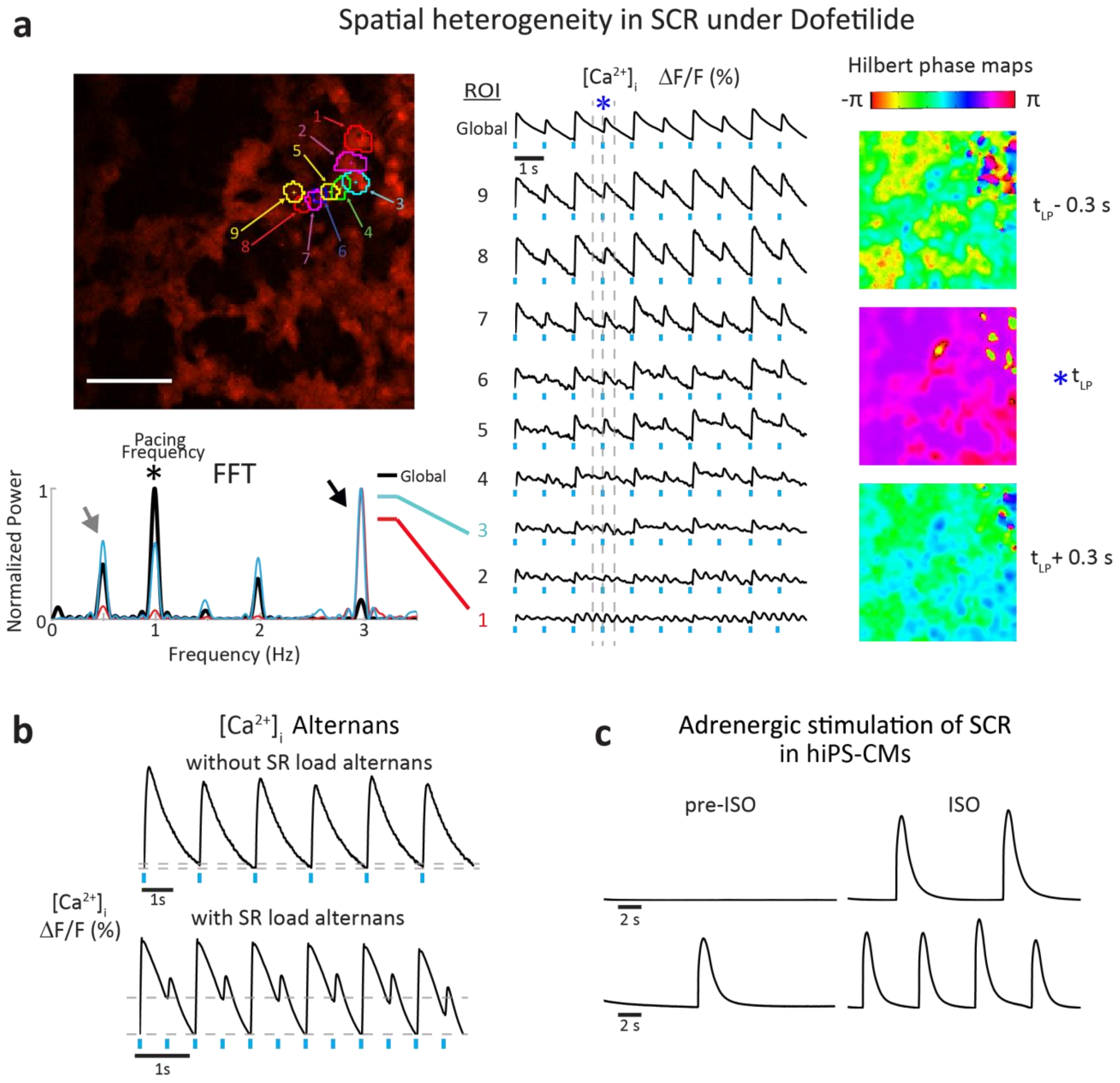

Supplementary Figure 6: Capturing sub-cellular spontaneous Ca^{2+} release (SCR) events. The recording of SCR events and other calcium instabilities within multicellular samples can be easily performed using OptoDyCE. **(a)** Example of spatial heterogeneity with localized spontaneous calcium release in a ChR2-CM sample treated with 0.02 μM dofetilide, when paced at 1 Hz using a 0.539 mW/mm^2 10 ms light pulses. Traces from 9 outlined ROIs (top left; scale bar 100 μm), along with the global trace, exhibit abnormal pacing behavior (center). Deviation from the global signal, progressing from ROI 9 to ROI 1, can be seen in both trace morphology and in the frequency domain (FFT plot left), where the 1Hz pacing frequency is indicated by an asterisk; a strong low frequency component at 0.5 Hz is due to alternans (arrow), while the observed sub-cellular SCR results in a higher frequency component (arrow) at 3 Hz. Phase maps (Hilbert transform, right) were used to identify localized intracellular Ca^{2+} waves due to SCR; the three panels, corresponding to the grey dashed lines in the recorded traces, show the instantaneous phase 300 ms prior to the optical stimulus (t_{LP} – 0.3s), during the optical stimulus (t_{LP}), and 300 ms after the optical stimulus (t_{LP} + 0.3s). **(b)** OptoDyCE can identify Ca^{2+} alternans with and without SR load alternation:

example trace from "spark"-hiPSC-CMs (top) shows subtle alternans without SR load alternation when paced at 0.5 Hz with a 1.51 mW/mm^2 5 ms pulses, while an example trace from a ChR2-CMs sample paced at 2 Hz with a 0.455 mW/mm^2 10 ms pulse shows prominent SR load alternation (dashed lines). **(c)** Calcium-mediated pacing activity was induced by 1 μM isoproterenol (10 min) in quiescent hiPS-CMs (top) and rate of pacing was accelerated in spontaneously beating hiPS-CMs (bottom).

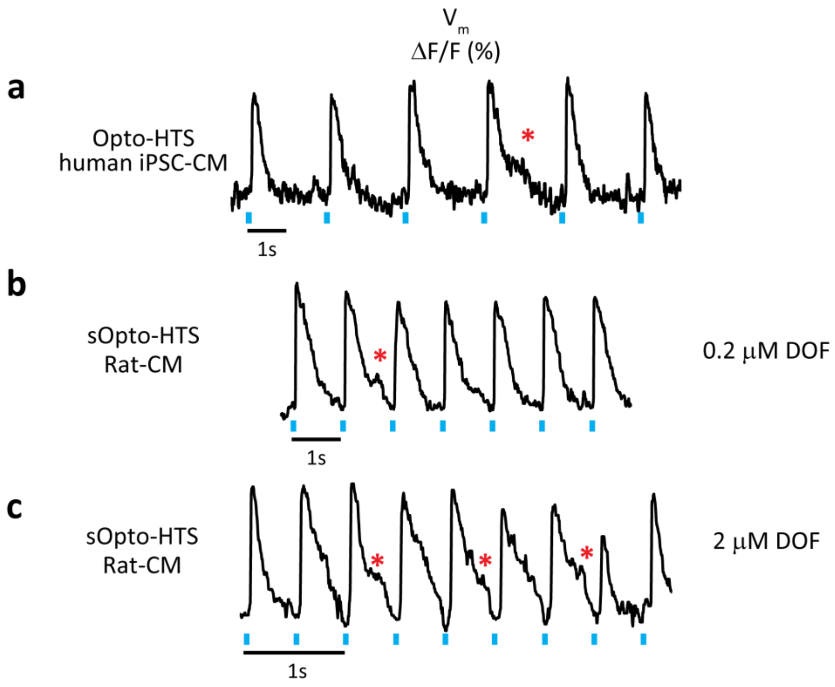

Supplementary Figure 7: Abnormalities in action potential morphology captured by OptoDyCE. (a) Intrinsic variability, including EADs (red asterisks), observed in this ChR2-hiPSC-CM sample, paced at 0.5 Hz pacing. Drug-induced AP variation in sOptoHTS samples treated with 0.2 μM dofetilide **(b)** and 2 μM dofetilide **(c)** paced at 1 Hz and 2 Hz, respectively. Lower doses of dofetilide create sporadic EADs (red asterisks), while higher doses yield large temporal variability in AP morphology (in addition to spatial variability quantified in **Fig. 6**), with increased instances of EADs.

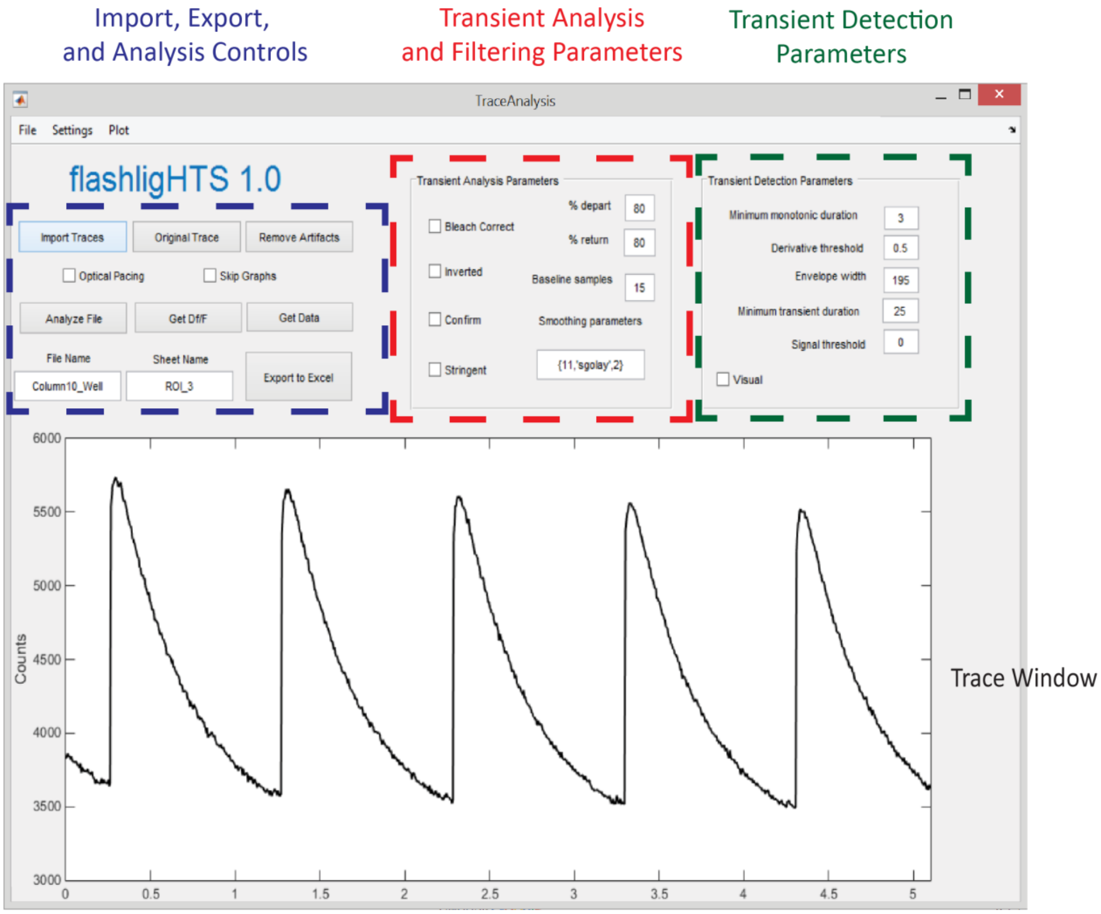

Supplementary Figure 8: flashligHTS analysis software. A snapshot of the custom-developed automated analysis software is shown.

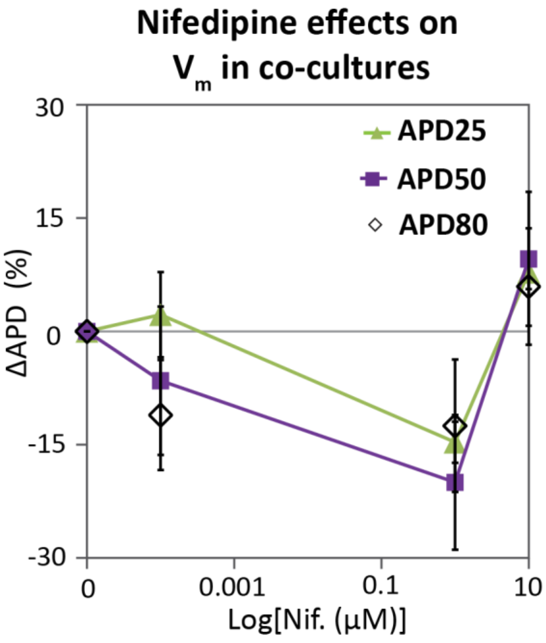

Supplementary Figure 9: Drug action in standard TCU co-cultures. Shown is the effect of nifedipine on APD in standard co-cultures based on the TCU concept, when the "spark" cells are not "sprinkled" at a later point but rather mixed uniformly at the time of plating of the CMs. Comparing with **Fig. 4a**, all three cases qualitatively capture the action of nifedipine on APD, but the sOptoHTS with "sprinkling" is the simplest, most modular and attractive for industrial application.

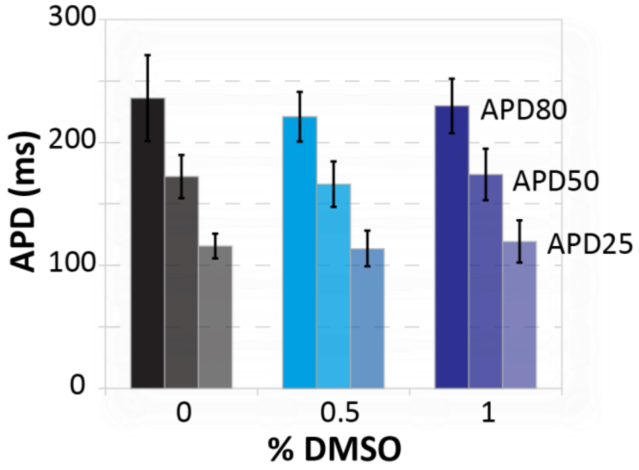

Supplementary Figure 10: Effect of DMSO on APD. CM-ChR2 cells were dosed with 0, 0.5, and 1 % DMSO in Tyrode's solution to assess the effect of DMSO on APD. Within the used concentrations to administer drugs and/or dyes (in all cases <1%), DMSO was not seen to affect the electrophysiological measurements.

Supplementary Tables

Supplementary Table 1.

State-of-the art industry-employed high-throughput electrophysiological measurement approaches

System	Description	PROS	CONS
**IonWorks** *Molecular Devices* *2-7*	***Actuation:*** Electrical ***Sensing:*** Population patch clamp	• Closest to classic ion channel characterization<br>• Fast readout<br>• Dynamic stimulation available	• Limited throughput/expandability<br>• Limited spatial resolution<br>• Contact-requiring<br>• Not robust (needs "well behaved" cell lines; not applicable to any primary cells or mini-tissues)<br>• No calcium/contractility measurements<br>• High complexity; custom plates<br>• High cost
**Maestro Multichannel Electrode Array** *(MEAs) Axion Biosystems* *2, 8-11*	***Actuation:*** Electrical ***Sensing:*** Local field potential	• Label-free<br>• Long-term recording possible<br>• Fast readout<br>• Dynamic stimulation available	• Limited throughput/expandability<br>• Limited spatial resolution<br>• Contact-requiring<br>• Not robust (not applicable to mini-tissues)<br>• No direct measurements of action potentials, calcium or contractility<br>• High complexity; custom plates
**xCELLigence** *Acea Biosciences* *8, 9, 12, 13*	***Actuation:*** Electrical ***Sensing:*** Impedance	• Label-free<br>• Long-term recording possible	• Limited throughput/expandability<br>• Tracks only slow processes<br>• Limited spatial resolution<br>• Contact-requiring<br>• Not robust (not applicable to mini-tissues)<br>• No direct measurements of action potentials, calcium or dynamic contractions<br>• High complexity; custom plates<br>• High cost
**FLIPR** *Molecular Devices* 3, 7, 14, 15	***Actuation:*** Chemical ***Sensing:*** Optical (fluorescence)	• Highly parallel<br>• Contactless<br>• Optical readout	• No dynamic stimulation<br>• Slow readout<br>• No spatial information<br>• Not robust (not applicable to mini-tissues)<br>• No direct measurements of action potentials or contractility<br>• High complexity; custom plates<br>• High cost
**FDSS/µCell** *Hamamatsu* *14, 16*	***Actuation:*** Chemical (or electrical field) ***Sensing:*** Optical (fluorescence)	• Highly parallel<br>• Contactless<br>• Optical readout<br>• Dynamic stimulation available	• Limited dynamic stimulation<br>• Relatively slow readout (typical < 5fps)<br>• No spatial information (low SNR)<br>• Not robust (not applicable to mini-tissues)<br>• No direct measurements of action potentials or contractility<br>• High complexity; custom plates<br>• High cost

Supplementary Notes

Supplementary Note 1

The pre-clinical process of drug discovery and testing involves multiple stages with different objectives and different suitable experimental systems and assays. For cardiac electrophysiology applications, the top, **high-throughput** type of assays should yield fast, massively-parallel coverage of a large number of compounds and combinations in order to eliminate high-risk items. Currently, there are no HT electrophysiological assays in place. As illustrated in **Fig. 1**, OptoDyCE can elevate electrophysiological testing to HT-status, if the experimental model is also HT-compatible. Cardiomyocytes and small engineered tissues that can be cultured fit this paradigm. Despite recognized current problems with human iPSC-CMs, namely immaturity and variability[17], we argue that there may not be better alternatives when HT-format assays are considered.

Secondary filters in the drug testing process for cardiac electrophysiology can be provided by medium to low throughput assays designed to test also tissue-level / conduction properties. Engineered tissue equivalents may be suitable, but native cardiac tissue (animal or human-derived) provides a superior testing platform for assessing conduction abnormalities in this lower-throughput format (**Fig. 1**). Native tissue is not a suitable experimental target for the HT-level screening tests for several reasons:

- Source and relevance: The excitement with stem-cell derived myocytes (esp. iPSC-CMs) is based on the idea that drug testing can become patient-specific. This cannot be achieved with native human heart tissue (i.e. patient heart biopsies are invasive and not desirable). There is a limited availability of donor human heart tissue, which varies a lot in state, patient age, drug and disease history, making it unsuitable for HT screening purposes.
- Spatial constraints: HT inevitably means scaling-down the sample size, dramatically, to increase throughput within the same plate format. If native tissue is to be used (animal or human), it must be chopped up to very tiny pieces (in the sub-millimeter range) in order to provide independent samples and to make it amenable to HT-format measurements. This presents problems with handling and viability. An interesting alternative is to use a "patterned" drug application (via microfluidics) within a bigger tissue sample[18]. In the case of cardiac tissue, inherent electrical coupling between the cells and inherent spatial variations present a problem for independent readouts.
- Viability and sample stability: Cultured cell systems are inherently better suited for industrial scale handling. They are easier to modify genetically in a consistent manner; they are functionally more stable over time than organotypic cultures, for example, which show rapid de-differentiation and loss of function, despite ongoing efforts to stabilize them (i.e. efforts to produce long-term cultured tissue slices)[19].

Finally, the preclinical process requires testing of systems-level effects, which needs to be done in live animals and is low-throughput in nature. The main objective is to assess the drug's metabolism and its action at the system's level (including other organs), and longer-term effects that may reveal unexpected new risks.

All-optical methods are compatible with experimental models across these stages, but they are most impactful at the high-throughput level, which is the focus of this study.

Supplementary References

1. Fermini, B. et al. A New Perspective in the Field of Cardiac Safety Testing through the Comprehensive In Vitro Proarrhythmia Assay Paradigm. *J Biomol Screen* (2015).
2. Dunlop, J., Bowlby, M., Peri, R., Vasilyev, D. & Arias, R. High-throughput electrophysiology: an emerging paradigm for ion-channel screening and physiology. *Nat Rev Drug Discov* **7**, 358-368 (2008).
3. Elkins, R.C. et al. Variability in high-throughput ion-channel screening data and consequences for cardiac safety assessment. *Journal of Pharmacological and Toxicological Methods* **68**, 112-122 (2013).
4. Finkel, A. et al. Population Patch Clamp Improves Data Consistency and Success Rates in the Measurement of Ionic Currents. *Journal of Biomolecular Screening* **11**, 488-496 (2006).
5. Fertig, N. & Farre, C. Renaissance of ion channel research and drug discovery by patch clamp automation. *Future Medicinal Chemistry* **2**, 691-695 (2010).
6. Schroeder, K., Neagle, B., Trezise, D.J. & Worley, J. IonWorks™ HT: A New High-Throughput Electrophysiology Measurement Platform. *Journal of Biomolecular Screening* **8**, 50-64 (2003).
7. Terstappen, G.C., Roncarati, R., Dunlop, J. & Peri, R. Screening technologies for ion channel drug discovery. *Future Medicinal Chemistry* **2**, 715-730 (2010).
8. Leyton-Mange, J. & Milan, D. Pluripotent Stem Cells as a Platform for Cardiac Arrhythmia Drug Screening. *Curr Treat Options Cardio Med* **16**, 1-18 (2014).
9. Khan, J.M., Lyon, A.R. & Harding, S.E. The case for induced pluripotent stem cell-derived cardiomyocytes in pharmacological screening. *British Journal of Pharmacology* **169**, 304-317 (2013).
10. Hescheler, J. et al. Determination of electrical properties of ES cell-derived cardiomyocytes using MEAs. *Journal of Electrocardiology* **37, Supplement**, 110-116 (2004).
11. Halbach, M.D., Egert, U., Hescheler, J. & Banach, K. Estimation of Action Potential Changes from Field Potential Recordings in Multicellular Mouse Cardiac Myocyte Cultures. *Cellular Physiology and Biochemistry* **13**, 271-284 (2003).
12. Guo, L. et al. Refining the Human iPSC-Cardiomyocyte Arrhythmic Risk Assessment Model. *Toxicological Sciences* **136**, 581-594 (2013).
13. Himmel, H.M. Drug-induced functional cardiotoxicity screening in stem cell-derived human and mouse cardiomyocytes: Effects of reference compounds. *Journal of Pharmacological and Toxicological Methods* **68**, 97-111 (2013).
14. Eggeling, C., Brand, L., Ullmann, D. & Jäger, S. Highly sensitive fluorescence detection technology currently available for HTS. *Drug Discovery Today* **8**, 632-641 (2003).
15. Schroeder, K.S. & Neagle, B.D. FLIPR: A New Instrument for Accurate, High Throughput Optical Screening. *Journal of Biomolecular Screening* **1**, 75-80 (1996).
16. Poul, E.L. et al. Adaptation of Aequorin Functional Assay to High Throughput Screening. *Journal of Biomolecular Screening* **7**, 57-65 (2002).
17. Knollmann, B.C. Induced pluripotent stem cell-derived cardiomyocytes: boutique science or valuable arrhythmia model? *Circ Res* **112**, 969-976 (2013).
18. Chang, T.C. et al. Parallel microfluidic chemosensitivity testing on individual slice cultures. *Lab Chip* **14**, 4540-4551 (2014).
19. Brandenburger, M. et al. Organotypic slice culture from human adult ventricular myocardium. *Cardiovasc Res* **93**, 50-59 (2012).
